# Neonatal Tactile Stimulation Alters Behaviors in Heterozygous Serotonin Transporter Male Rats: Role of the Amygdala

**DOI:** 10.3389/fnbeh.2020.00142

**Published:** 2020-08-12

**Authors:** Karine Roversi, Carolina Buizza, Paola Brivio, Francesca Calabrese, Michel M. M. Verheij, Caren T. D. Antoniazzi, Marilise E. Burger, Marco A. Riva, Judith R. Homberg

**Affiliations:** ^1^Department of Physiology and Pharmacology, Health Sciences Center, Federal University of Santa Maria, Santa Maria, Brazil; ^2^Department of Pharmacological and Biomolecular Sciences, Università Degli Studi di Milano, Milan, Italy; ^3^Department of Cognitive Neuroscience, Donders Institute for Brain, Cognition, and Behavior, Radboud University Medical Center, Nijmegen, Netherlands; ^4^International Centre for Neurotherapeutics, Dublin City University, Dublin, Ireland

**Keywords:** neonatal period, tactile stimulation, anxiety, Bdnf, serotonin transporter knockout, amygdala

## Abstract

The serotonin transporter (SERT) gene, especially the short allele of the human serotonin transporter linked polymorphic region (5-HTTLPR), has been associated with the development of stress-related neuropsychiatric disorders. In line, exposure to early life stress in SERT knockout animals contributes to anxiety- and depression-like behavior. However, there is a lack of investigation of how early-life exposure to beneficial stimuli, such as tactile stimulation (TS), affects later life behavior in these animals. In this study, we investigated the effect of TS on social, anxiety, and anhedonic behavior in heterozygous SERT knockouts rats and wild-type controls and its impact on gene expression in the basolateral amygdala. Heterozygous SERT^+/–^ rats were submitted to TS during postnatal days 8–14, for 10 min per day. In adulthood, rats were assessed for social and affective behavior. Besides, brain-derived neurotrophic factor (Bdnf) gene expression and its isoforms, components of glutamatergic and GABAergic systems as well as glucocorticoid-responsive genes were measured in the basolateral amygdala. We found that exposure to neonatal TS improved social and affective behavior in SERT^+/–^ animals compared to naïve SERT^+/–^ animals and was normalized to the level of naïve SERT^+/+^ animals. At the molecular level, we observed that TS *per se* affected Bdnf, the glucocorticoid-responsive genes Nr4a1, Gadd45β, the co-chaperone Fkbp5 as well as glutamatergic and GABAergic gene expression markers including the enzyme Gad67, the vesicular GABA transporter, and the vesicular glutamate transporter genes. Our results suggest that exposure of SERT^+/–^ rats to neonatal TS can normalize their phenotype in adulthood and that TS *per se* alters the expression of plasticity and stress-related genes in the basolateral amygdala. These findings demonstrate the potential effect of a supportive stimulus in SERT rodents, which are more susceptible to develop psychiatric disorders.

## Introduction

Vulnerability to psychiatric disorders is thought to be caused by complex interactions between genes and the environment particularly when the interactions take place early in life (Caspi et al., 2010). The serotonin transporter linked polymorphic region (5-HTTLPR) serves as a model for gene-environment interactions, with the short (s) 5-HTTLPR allele playing an important role in the sensitivity to the environment (Homberg and Lesch, 2011; Homberg et al., 2016). In rodents, these behavioral conditions can be evaluated in serotonin transporter (SERT) knockout rats (Homberg et al., 2007). Heterozygous SERT knockout animals (SERT^+/–^) are generally suggested to be most comparable to the human short 5-HTTLPR allele from a gene-dose dependent point of view. Although some studies could not confirm an interaction effect of SERT genotype and early life adversities (see review: Houwing et al., 2017), other studies have shown a significant influence of the SERT^+/–^ genotype on behaviors putatively related to psychiatric disorders (Olivier et al., 2008; Bartolomucci et al., 2010; van der Doelen et al., 2013; Houwing et al., 2019).

SERT knockout animals present enhanced anxiety- and depression-like symptoms (Homberg et al., 2008; Schipper et al., 2015, 2019; Verheij et al., 2018) and most studies examined the increased sensitivity to negative environmental stimuli in SERT knockout rodents. However, a few studies demonstrated that these animals are also very sensitive to positive environmental stimuli such as psychostimulants, conditioned reward, co-housing with a female, and environmental enrichment (Homberg and Lesch, 2011; Nonkes et al., 2012; Kastner et al., 2015; Homberg et al., 2016; Rogers et al., 2017). This is well in line with the differential susceptibility theory from the field of developmental psychology, which postulates that “plastic” individuals, due to (5-HTTLPR s/s) genotype, show increased sensitivity to environmental stimuli, both adverse and supportive ones (Belsky et al., 2009; van Ijzendoorn et al., 2012).

Early life is a critical phase for central nervous system development, during which plasticity levels are high and the brain is very sensitive to environmental influences. Thus far, no studies have addressed the effect of an early life supportive environment on later life behavior in SERT^+/–^ rodents. Neonatal handling is an environmental treatment used to study behavioral mechanisms and neurobiological alterations in rodents (Denenberg, 1964). The handling consists of separating the pups from the mothers for a short period and performing some intervention, such as tactile stimulation (TS; Daskalakis et al., 2009). Neonatal TS is a procedure applied during developmental periods mimicking nonspecific maternal stimulation such as licking and grooming of pups. It has emerged as an efficient tool to improve the behavior by altering brain organization and enhancing hippocampus neurogenesis (Guerrero et al., 2016) and neuroplasticity (Richards et al., 2012). TS decreases anxiety-like behaviors (Río-Alamos et al., 2015), and prevents the negative effects of stress (Boufleur et al., 2013) and the development of depressive-like behaviors (Freitas et al., 2015). These findings raise the possibility that TS has the potential to modify the anxiety- and depression-related phenotypes of SERT^+/–^ animals.

The TS mechanism in the brain is still unclear, but evidence has suggested that TS affects the hypothalamic-pituitary-adrenal (HPA) axis and brain neurotrophic factors such as brain-derived neurotrophic factor (Bdnf; Antoniazzi et al., 2017; Roversi et al., 2019). Further, the GABA and glutamate systems are likely targets. The HPA-axis mediates stress responses through glucocorticoids release from the adrenal cortex. The programming of the HPA-axis is influenced by stress, leading to glucocorticoid receptor (Nr3c1) dysregulation and changes in the expression of glucocorticoid-inducible genes, including the co-chaperone FK506-binding protein 51 (Fkbp5) and the transcription factor Nr4a1 (Kember et al., 2012; van der Doelen et al., 2014). Indeed, the expression of Fkbp5 was found to be altered in peripheral blood cells of patients suffering from psychiatric disorders following early life adversities (Klengel et al., 2013). Furthermore, altered stress-related behavior in adulthood has been associated with changes in Nr4a1 gene expression in the hippocampus of mice (Kember et al., 2012). Also, the growth arrest and DNA-damage-inducible beta (Gadd45β), a glucocorticoid-responsive gene responsible for actively demethylating gene promoter regions, was found to be increased in the hippocampus of rats treated with the multitarget antidepressant vortioxetine and exposed to acute stress (Brivio et al., 2019). Finally, negative experiences in early life seem to affect the levels of Gadd45β, yet also overexpression of this gene has been observed in the amygdala of psychiatric patients (Gavin et al., 2012; Blaze and Roth, 2013). Thus, whether Gadd45β is also responsive to positive stimuli in early life remains to be determined.

Bdnf is a molecule that promotes the development and survival of neurons and is present in high amounts in the basolateral amygdala. Changes in Bdnf levels in this area are associated with stress-dependent learning and important behavioral changes related to fear and depression (Williams et al., 2006; Schulte-Herbruggen et al., 2006). While TS increased Bdnf hippocampus levels and improved memory and anxiety behaviors in normal and depressive-like animals (Antoniazzi et al., 2017; Roversi et al., 2019), transgenic overexpression of Bdnf in the amygdala facilitated the development of anxiety-related behaviors (Govindarajan et al., 2006), thus showing the importance of the modulatory effect of Bdnf in the brain. Neonatal handling also affects the GABAergic system, as demonstrated by increased GABA interneuron density in the lateral amygdala (Giachino et al., 2007). Moreover, there is an important link between the GABAergic and glutamatergic systems in animal models of depression and different stress-related psychiatric disorders (Garcia-Garcia et al., 2009; Luscher et al., 2011), such as depression, which is characterized by an excitation-inhibition imbalance (Sanacora et al., 2004). Furthermore, expression levels of Gad67, the enzyme that converts glutamate in GABA, are decreased in psychiatric disorders (Fatemi et al., 2005). Conversely, positive stimuli like environmental enrichment that can buffer the negative effects of stress also alter GABAergic signaling in the amygdala (Sampedro-Piquero et al., 2016). Finally, there is evidence that Bdnf affects the excitation-inhibition balance, providing a putative pathway through which positive environmental stimuli may ameliorate stress-induced excitation-inhibition imbalances (Oh et al., 2016).

In this study we sought out to determine the effect of a supportive environment, that is TS, during the postnatal day (PND) 8–14. This period of life was chosen based on a previous study of Antoniazzi et al. (2017), in which the most beneficial effects of TS have been observed during this period. Furthermore, in rats, brain development paces extremely fast during the first weeks of life. Neurogenesis is completed on PND 15 (Rice and Barone, 2000; Babikian et al., 2010), the astrocytes undergo to a rapid period of maturation, with a peak on PND 11–16 (Catalani et al., 2002), and the critical period of synaptogenesis peaks during week 2 after birth (Semple et al., 2013). As readouts we focused on social behavior, anhedonia, and anxiety in SERT^+/–^ and wild-type control rats in adulthood. Additionally, to understand the underlying mechanisms we assessed mRNA expression levels of Bdnf, glucocorticoid (Nr3c1) and mineralocorticoid (Nr3c2) receptors, and glucocorticoid-responsive genes in the basolateral amygdala. We also focused on components of the GABAergic and glutamatergic systems, by analyzing Gad67, vesicular GABA transporter (Vgat), parvalbumin, and vesicular glutamate transporter (Vglut) gene expression.

## Materials and Methods

### Animal and Procedures

**Figure 1:**
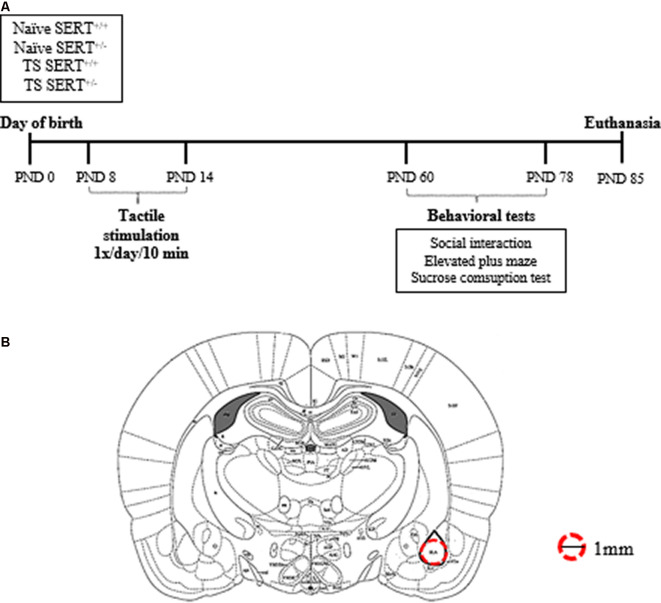
Schematic representation of the experimental paradigm performed in heterozygous serotonin transporter knockout (SERT^+/–^) and wild-type (SERT^+/+^) male rats exposed daily to tactile stimulation (TS) from postnatal day (PND) 8–14 or a control treatment (no TS-naïve; **A**). Diagrams represent the location where basolateral amygdala punches were taken for molecular analyses (adapted from Paxinos and Watson brain Atlas; **B**).

Eight wild-type and eight SERT^+/–^ (Slc6a41Hubr) pregnant rats were used. They were derived by crossing heterozygous breeding animals. Dams were checked daily for pups’ delivery, and the day of birth was set as postnatal day (PND) 0. On PND8–14, male pups from each litter were assigned to one of the two experimental groups: neonatal TS or not (no TS, naïve), and the pups were marked with a non-toxic colored marker for identification purposes. At PND21, pups were weaned, and ears were punched for identification and genotyping (Homberg et al., 2007). The animals were housed in two animals per cage, in standard polypropylene cages with saw-dust bedding and water and food *ad libitum*, in a temperature (21 ± 1°C) and humidity-controlled room (45%–60% relative humidity), with a 12:12 h light/dark cycle (lights on at 7:00 AM). The experimental procedures were approved by the Committee for Animal Experiments of the Radboud University Nijmegen, The Netherlands, and all efforts were made to minimize animal suffering and to reduce the number of animals used. Only male SERT^+/–^ rats were used in this study. At PND 60, the males from naïve and TS were subdivided between treatment and genotype [wild-type (SERT^+/+^) or heterozygous (SERT^+/–^)] groups, resulting in the following groups: naïve SERT^+/+^ (*n* = 9); naïve SERT^+/–^ (*n* = 7); TS SERT^+/+^ (*n* = 9); TS SERT^+/–^ (*n* = 9; Figure 1A). All tests were performed in the dark phase.

### Neonatal Tactile Stimulation

Neonatal TS was applied from PND 8 to PND 14, between 10 AM and 2 PM. Pups were removed from the nest, gently held by the experimenter, and stroked with the index finger on the dorsal surface, in the rostral-caudal direction for 10 min, once a day. At the end of the TS, pups were returned to their litters. The naïve group (no TS) remained in their nest without any touch by human hands (Freitas et al., 2015; Antoniazzi et al., 2017).

### Behavioral Procedures

#### Social Interaction Test

The social interaction test protocol is based on social interest and interaction with an unfamiliar animal. The arenas consisted of a black floor and transparent walls (45 cm × 50 cm). Before the test, animals were put individually in the chamber for a 1-h habituation session and immediately after habituation, two rats from different cages with the same genotype and manipulation were put together in the arena and the behavior was video recorded for 15 min. The recorded behavior was measured as social contact (time animals spent in body-contact, sniffing, grooming), social interest (time animal was following or approaching), social undergoing (passive behavior: when the rat’s partner was mounting, sniffing, pinning, attacking the rat), and non-social behavior (time animal was solitary). One observer blind to the test subjects’ genotype and manipulation, manually scored video recordings by using Boris v.7. Both animals were equally observed. By using a reliability analysis feature in Boris (Behavioral Observation Research Interactive Software), the percentages of agreement between two observers were calculated and resulted in 78% of agreement (data not shown).

#### Elevated Plus-Maze

This test is based on the innate fear rodents have for open and elevated spaces and was performed as described by de Jong et al. (2006). The apparatus consisted of a plus-shaped platform elevated 50 cm from the floor. Two opposite arms (50 cm × 10 cm) were enclosed by 40 cm high walls whereas the other two arms had no walls. The four arms had at their intersection a central platform, which gave access to any of the four arms. At the beginning of each test, the rat was placed on the central platform facing an open arm. The movements and position of the animals were recorded and processed afterward using EthoVision XT (Noldus Information Technology, The Netherlands). Entries were counted when all four paws were placed in one of the arms. Data were expressed as the mean percentage of the time spent on open arms [(time on open arms/300 s) × 100%], the mean of the entries number into open arms [(entries on open arms/total entries) × 100%] and the time spent on closed arms (s). The mean of the time spent (s) on and number of entries to open arms were used as the standard anxiety indices. Locomotor activity was expressed as the total distance moved (cm) by the animal in the entire maze.

#### Sucrose Consumption

Anhedonia is typically measured using the sucrose consumption test in which the animals have in their homecage a free choice between a bottle with water and a bottle with a sucrose solution. A decrease in sucrose intake and/or preference is considered as a measure of anhedonia (Willner et al., 1987). Animals were housed individually and habituated to the two-bottle paradigm by offering them water in two plastic drinking bottles, one on each side, for a total of 3 days. After the third day of the habituation period, the sucrose test started. Animals were presented with two bottles, one containing water and the other one containing a 3% sucrose solution. Fluid consumption (g) was measured at 2 h, 5 h, and 24 h, and body weight was measured daily (g). Both measures were used to calculate sucrose preference (sucrose intake in ml divided by total intake × 100%) and the intake in grams relative to body weight in Kg (intake in grams divided by body weight in grams/1,000; adapted from Olivier et al., 2008).

#### Tissue Collection and Preparation

One week after the last day of the sucrose test, the animals were euthanized by decapitation within 10 s. Immediately after the decapitation, the brains were isolated and frozen on dry ice in an aluminum foil and stored in a −80° C. The brains were prepared in 200 μm thick coronal slices in a cryostat (−11°C) to obtain punches of basolateral amygdala bilaterally (Bregma ≈ −2.28: −3.30 mm; Interaural ≈ −6.72: −5.80 mm; anterior-posterior ≈2.8 posterior to bregma; DV ≈6.5 from skull surface) using a 1.00 mm brain puncher (Figure 1B). The brain puncher was cleaned with alcohol after each punch. The punches were used for RNA isolation.

##### RNA Preparation and Gene Expression Analysis by Quantitative Real-Time RT-PCR

Total RNA was isolated from the basolateral amygdala using PureZol RNA isolation reagent (Bio-Rad Laboratories S.r.l.; Segrate, Italy) following the manufacturer’s instructions. The RNA concentration was then quantified by spectrophotometric analysis (OD 260/280 1.8 < ratio <2). Afterward, the samples were prepared for real-time polymerase chain reaction (RT-PCR) to measure the mRNA expression of total Bdnf, Bdnf long 3’ UTR, Bdnf isoforms IV and VI, Nr3c1, Nr3c2, Nr4a1, Gadd45β, Fkbp5, Gad67, Pvalb, Vgat, Vglut. After RNA isolation, an aliquot of each sample was treated with DNAse to avoid contamination by DNA. The samples were then prepared for the analyses by TaqMan qRT-PCR instrument (CFX384 real-time system, Bio-Rad Laboratories S.r.l.) using the iScript one-step RT-PCR kit for probes (Bio-Rad Laboratories S.r.l.). The primers and probes sequences used were acquired from Eurofins MWG-Operon and Life Technologies and are shown in Table 1. The samples (10 ng/ul) were run in a 348-well format in triplicates as multiplexed reactions with the normalizing internal control 36b4. Thermal cycling started with incubation at 50°C for 10 min (RNA retrotranscription), and 95°C for 5 min (TaqMan polymerase activation). After this step, 39 cycles of PCR were performed. Each PCR cycle consisted of heating the samples at 95°C for 10 s to facilitate the melting process and then at 60°C for 30 s for the annealing and extension reactions. A comparative cycle threshold (Ct) method was used to calculate the relative target gene expression by applying the 2^(−ΔΔCT)^ method (Livak and Schmittgen, 2001).

**Table 1:** (a) Sequences of primers, reverse primers, and probes used in real-time polymerase chain reaction (RT-PCR) analyses and purchased from Eurofins MWG-Operon.

**(a) Gene**	**Forward primer**	**Reverse primer**	**Probe**
Total Bdnf	AAGTCTGCATTACATTCCTCGA	GTTTTCTGAAAGAGGGACAGTTTAT	TGTGGTTTGTTGCCGTTGCCAAG
Nr3c1	GAAAAGCCATCGTCAAAAGGG	TGGAAGCAGTAGGTAAGGAGA	AGCTTTGTCAGTTGGTAAAACCGTTGC
Nr3c2	TCGCTTTGAGTTGGAGATCG	ACGAATTGAAGGCTGATCTGG	AGTCTGCCATGTATGAACTGTGCCA
Pvalb	CTGGACAAAGACAAAAGTGGC	GACAAGTCTCTGGCATCTGAG	CCTTCAGAATGGACCCCAGCTCA
Vgat	ACGACAAACCCAAGATCACG	GTAGACCCAGCACGAACATG	TTCCAGCCCGCTTCCCACG
Gad67	ATACTTGGTGTGGCGTAGC	AGGAAAGCAGGTTCTTGGAG	AAAACTGGGCCTGAAGATCTGTGGT
Vglut	ACTGCCTCACCTTGTCATG	GTAGCTTCCATCCCGAAACC	CTTTCGCACATTGGTCGTGGACATT
Fkbp5	GAACCCAATGCTGAGCTTATG	ATGTACTTGCCTCCCTTGAAG	TGTCCATCTCCCAGGATTCTTTGGC
36b4	TTCCCACTGGCTGAAAAGGT	CGCAGCCGCAAATGC	
	AAGGCCTTCCTGGCCGATCCATC		
**(b) Gene**	**Accession number**	**Assay ID**	
Bdnf long 3’ UTR	EF125675	Rn02531967_s1	
Bdnf isoform IV	EF125679	Rn01484927_m1	
Bdnf isoform VI	EF125680	Rn01484928_m1	
Gadd45β	BC085337.1	Rn01452530_g1	
Nr4a1	BC097313.1	Rn01533237_m1	

*(b) Probes purchased from Life Technologies which did not disclose the sequence*.

### Data Analysis

Behavioral data were analyzed by two-way ANOVA followed by *post hoc* of Newman-keuls. Genotype (SERT^+/+^ vs. SERT^+/–^) and manipulation (naïve vs. TS) were assessed as independent variables. The level of significance was set at *P* < 0.05. For the molecular data two-way ANOVA followed by *post hoc* of Fisher’s protected least significant difference (PLSD) was used. Benjamini–Hochberg multiple testing corrections for false discovery rate (FDR) was applied and the significance was set to FDR adjusted *p*-value < 0.05. All statistical analyses were performed using the Statistics Software version 13.3 (Tulsa, OK, USA) and the graphs were made by GraphPad Prism version 7. Data are presented as means ± standard error (SEM). In the graphs of the molecular data, the group of naïve SERT^+/+^ is set at 100%.

## Results

### Behavioral Tests

#### Social Interaction Test

**Figure 2:**
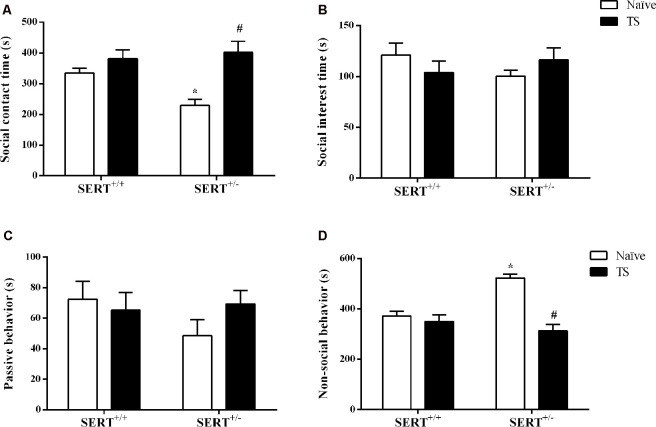
Social interaction test. The time spent on social contact **(A)**, social interest **(B)**, passive behavior **(C)** and non-social behavior **(D)** of heterozygous serotonin transporter knockout (SERT^+/–^) and wild-type (SERT^+/+^) male rats daily exposed to TS from postnatal day 8–14 or a control treatment (no TS-naïve). Data are represented as mean ± SEM. *Significantly different from naïve SERT^+/+^ rats, ^#^significantly different from naïve SERT^+/–^ rats (*p* < 0.05).

Our main readout in the social interaction test was the time spent on social interaction. For this parameter, we found a significant effect of TS (*F*_(1,30)_ = 10.16; *p* = 0.003). After *post hoc* testing, we found that naïve SERT^+/–^ rats spent less time on social contact (*p* = 0.014) compared to naïve SERT^+/+^ rats while in TS SERT^+/–^ rats this parameter was increased (*p* = 0.001) compared to naïve SERT^+/–^ rats (Figure 2A).

For non-social behavior a significant interaction between genotype and TS (*F*_(1,30)_ = 15.47; *p* = 0.0005) was found. *Post hoc* testing showed that naïve SERT^+/–^ rats spent more time on non-social contact (*p* = 0.0002) compared to naïve SERT^+/+^ rats, while TS SERT^+/–^ rats spent less time on non-social behavior (*p* = 0.0001) compared to naïve SERT^+/–^ rats (Figure 2D).

For social interest and passive undergoing behavior, we did not find any difference between groups (Figures 2B,C).

#### Elevated Plus-Maze Test

**Figure 3:**
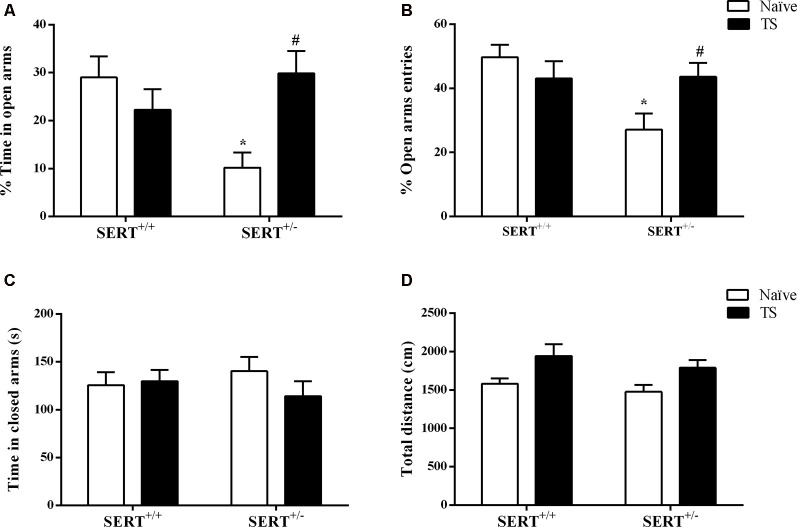
Elevated plus maze test. The mean time of spent in open arms (%; **A**), the mean of entries number in open arms (%; **B**), time spent in closed arms (s; **C**) and total distance traveled (cm; **D**) of heterozygous serotonin transporter knockout (SERT^+/–^) and wild-type (SERT^+/+^) male rats daily exposed to TS from postnatal day 8–14 or a control treatment (no TS-naïve). Data are represented as mean ± SEM. *Significantly different from naïve SERT^+/+^ rats, ^#^significantly different from naïve SERT^+/–^ rats (*p* < 0.05).

The standard readouts for anxiety as measured in the elevated plus-maze test involve the percentage time spent on the open arms and the percentage of entries onto the open arms. As shown in Figure 3A, two-way ANOVA showed that there was a significant interaction between genotype and TS (*F*_(1,30)_ = 9.29; *p* = 0.004) for the mean percentage of open arms time. *Post hoc* testing revealed that naïve SERT^+/–^ animals remained less time on the open arms compared to naïve SERT^+/+^ rats (*p* = 0.012) and TS SERT^+/–^ rats (*p* = 0.016; Figure 3A). For the relative number of open arm entries, a significant effect of genotype (*F*_(1,30)_ = 5.45; *p* = 0.026) and an interaction between genotype and TS (*F*_(1,30)_ = 6.05; *p* = 0.020) was observed. In particular, naïve SERT^+/–^animals showed fewer entries on to the open arm compared to naïve SERT^+/+^(*p* = 0.010) and TS SERT^+/–^ rats (*p* = 0.049; Figure 3B). These data show that TS ameliorated the anxiety observed in naïve SERT^+/–^ rats.

Regarding the time spent on closed arms, no statistical differences were observed (Figure 3C). For total time traveled, also no significant genotype or TS manipulation effect was found. The latter confirms that the differences seen in anxiety were not due to differences in locomotor behavior (Figure 3D).

#### Sucrose Consumption Test

**Figure 4:**
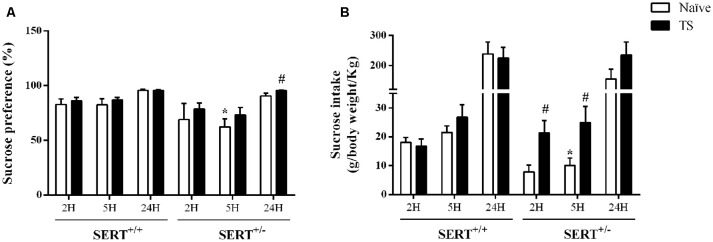
Sucrose consumption test. Sucrose preference **(A)** and sucrose intake **(B)** in heterozygous serotonin transporter knockout (SERT^+/–^) and wild-type (SERT^+/+^) male rats daily exposed to TS from postnatal day 8–14 or a control treatment (no TS-naïve). Data are represented as mean ± SEM. *Significantly different from naïve SERT^+/+^ rats, ^#^significantly different from naïve SERT^+/–^rats (*p* < 0.05).

As shown in Figure 4A, there was a significant effect of genotype (*F*_(1,30)_ = 8.19; *p* = 0.007) for sucrose preference over 5 h. Naïve SERT^+/–^ rats showed a reduction in sucrose preference after 5 h of exposure to the water and sucrose solutions (−25%; *p* = 0.049; Newman-Keuls) compared to the naïve SERT^+/+^ group. After 24 h of exposure, TS SERT^+/–^ rats increased their sucrose preference (+5%; *p* = 0.034) compared to naïve SERT^+/–^rats.

Figure 4B illustrates the sucrose intake data. We found a significant interaction between genotype and TS (*F*_(1,30)_ = 6.29; *p* = 0.018) effect over 2 h. A *post hoc* test revealed a trend towards a reduction (−58%; *p* = 0.053) in sucrose intake in naïve SERT^+/–^ rats after 2 h of exposure compared to naïve SERT^+/+^ rats, while TS SERT^+/–^ rats increased the sucrose intake after 2 h (+177%; *p* = 0.016) compared to naïve SERT^+/–^ rats.

Furthermore, for sucrose intake over 5 h, there was a TS effect (*F*_(1,30)_ = 6.24 *p* = 0.018). *Post hoc* testing showed that the sucrose intake in naïve SERT^+/–^ rats were lower than in naïve SERT^+/+^ rats (−36%; *p* = 0.048) while TS SERT^+/–^ rats showed an increase in sucrose intake after 5 h (+148%; *p* = 0.036) compared to naïve SERT^+/–^ rats. After 24 h of exposure, no significant differences were found in the sucrose intake.

### Molecular Results

#### Bdnf mRNA Expression Levels in the Basolateral Amygdala

**Figure 5:**
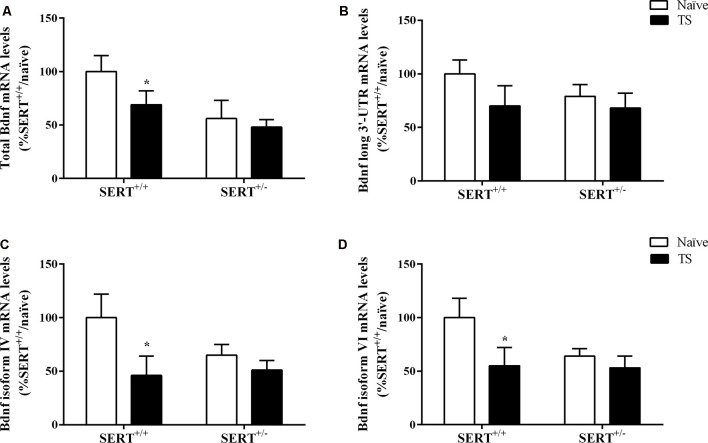
Total Bdnf **(A)**, Bdnf long 3’-UTR **(B)**, Bdnf exon IV **(C)** and exon VI **(D)** mRNA levels in the basolateral amygdala of heterozygous serotonin transporter knockout (SERT^+/–^), and wild-type (SERT^+/+^) male rats daily exposed to TS from postnatal day 8–14 or a control treatment (no TS-naïve). Data are expressed as a percentage of naïve SERT^+/+^ rats (set at 100%) and are presented as mean ± SEM. *Significantly different from naïve SERT^+/+^ rats (*p* < 0.05).

We initially investigated total Bdnf mRNA levels in the basolateral amygdala of naïve or TS SERT^+/–^ and SERT^+/+^ animals. We found a significant effect of TS (*F*_(1,31)_ = 6.053, *p* = 0.020) on total Bdnf mRNA levels. *Post hoc* analysis showed that the TS SERT^+/+^ group had a significant decrease of Bdnf mRNA levels compared to the naïve SERT^+/+^ group (−44%, *p* = 0.025; Figure 5A).

Based on this result, we decided to evaluate if TS could affect the expression of major Bdnf transcripts. In particular, we quantified the expression levels of the long 3’UTR Bdnf transcripts, associated with dendritic targeting of specific neurotrophin transcripts. Additionally, we measured Bdnf isoforms IV and VI. While isoform IV is localized in the soma, is an indication of altered neuronal activity (Pattabiraman et al., 2005), isoform VI is targeted to dendrites (Chiaruttini et al., 2008). Changes in isoform IV and VI have been associated with mood disorders (Molteni et al., 2010).

No significant differences were found for long 3’UTR Bdnf mRNA levels, indicating that there was no modulation of this pool of transcript on total Bdnf mRNA levels (Figure 5B). Two-way ANOVA revealed a significant effect of TS (*F*_(1,31)_ = 4.484, *p* = 0.043) for Bdnf isoform IV, with its mRNA levels being decreased in TS SERT^+/+^ animals (−54% vs. naïve SERT^+/+^, *p* = 0.024, Fisher PLSD) in comparison to the naïve counterpart (Figure 5C).

Regarding Bdnf exon VI, two-way ANOVA showed a similar result with a trend in the TS group (*F*_(1,31)_ = 3.794, *p* = 0.062). Indeed, we observed a decrease in Bdnf isoform VI levels in the TS SERT^+/+^ group (−44% vs. naïve SERT^+/+^, *p* = 0.035, Fisher PLSD; Figure 5D).

#### Mineralocorticoid and Glucocorticoid Receptor mRNA Expression Levels in the Basolateral Amygdala

We next investigated if mRNA expression levels of both corticosterone receptors, Nr3c1, and Nr3c2 could be affected by the SERT genotype or TS. Furthermore, the expression of glucocorticoid-responsive genes, including Nr4a1, Gadd45β, and the co-chaperone Fkbp5 were measured in the basolateral amygdala.

**Figure 6:**
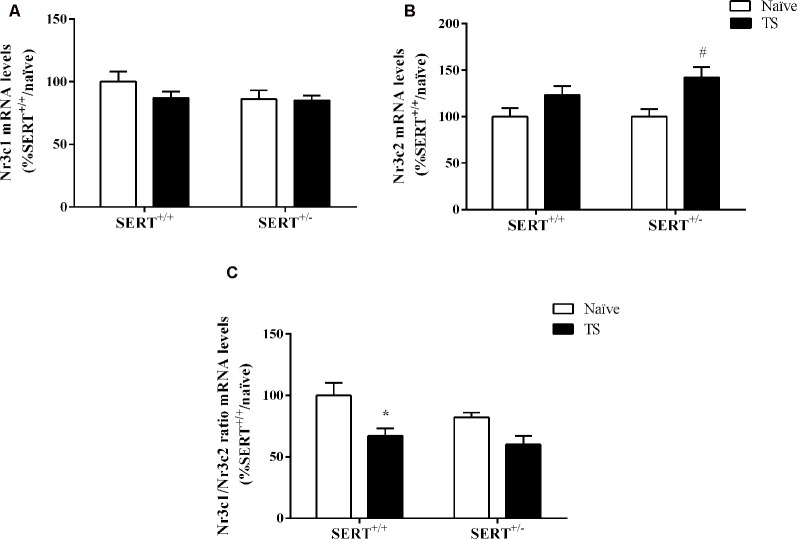
Glucocorticoid receptor (Nr3c1) **(A)**, mineralocorticoid receptor (Nr3c2) **(B)**, and the ratio between Nr3c1 and Nr3c2 **(C)** mRNA levels in the basolateral amygdala of heterozygous serotonin transporter knockout (SERT^+/–^), and wild-type (SERT^+/+^) male rats daily exposed to TS from postnatal day 8–14 or a control treatment (no TS-naïve). Data are expressed as a percentage of naïve SERT naïve rats (set at 100%) and are presented as mean ± SEM. *Significantly different from naïve SERT^+/+^ rats, ^#^significantly different from naïve SERT^+/–^ rats (*p* < 0.05).

Two-way ANOVA showed that TS significantly affected mRNA levels of Nr3c2 (*F*_(1,32)_ = 11.243, *p* = 0.002; Figure 6B). We observed that TS induced an increase of its mRNA levels specifically in SERT^+/–^rats (+42% vs. naïve SERT^+/–^, *p* = 0.003). We did not observe any change in Nr3c1 gene expression (Figure 6A). As a consequence, the Nr3c1/Nr3c2 ratio was significantly affected by TS (*F*_(1,32)_ = 11.541, *p* = 0.014). Fisher PLSD showed a decrease in this ratio in TS SERT^+/+^ rats compared to their naïve counterparts (−32% vs. naïve SERT^+/+^, *p* = 0.002; Figure 6C).

**Figure 7:**
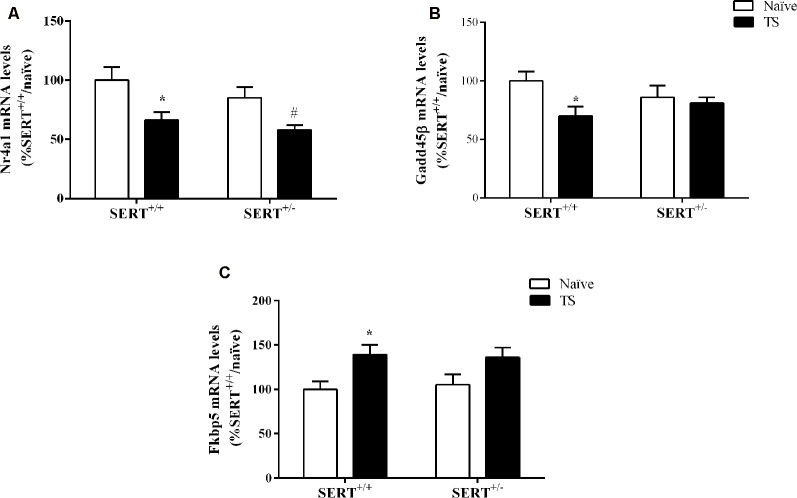
Nr4a1**(A)**, Gadd45β **(B)** and Fkbp5 **(C)** mRNA levels in the basolateral amygdala of heterozygous serotonin transporter knockout (SERT^+/–^), and wild-type (SERT^+/+^) male rats daily exposed to TS from postnatal day 8–14 or a control treatment (no TS-naïve). Data are expressed as a percentage of naïve SERT^+/+^ rats (set at 100%) and are presented as mean ± SEM. *Significantly different from naïve SERT^+/+^ rats, ^#^significantly different from naïve SERT^+/–^ rats (*p* < 0.05).

Two-way ANOVA of Nr4a1 mRNA levels revealed a significant effect of TS (*F*_(1,33)_ = 13.460, *p* = 0.0009; Figure 7A). *Post hoc* testing revealed a decrease in Nr4a1 mRNA levels both in TS SERT^+/+^ (−34%, *p* = 0.005) and TS SERT^+/–^ rats (−32%, *p* = 0.0008) compared to their naïve counterparts.

Gadd45β gene expression was significantly affected by TS (*F*_(1,33)_ = 4.887, *p* = 0.034, two-way ANOVA) and we found that TS induced a significant reduction of its mRNA levels in TS SERT^+/+^rats compared to naïve SERT^+/–^ rats (−28% vs. naïve SERT^+/+^, *p* = 0.009, Fisher PLSD; Figure 7B).

As shown in Figure 7C, we found a significant effect of TS on Fkbp5 gene expression (*F*_(1,33)_ = 10.377, *p* = 0.003, two-way ANOVA). Accordingly, we observed a significant increase in Fkbp5 mRNA levels due to TS in SERT^+/+^ rats compared to naïve SERT^+/+^ rats (+39% vs. naïve SERT^+/+^, *p* = 0.013, Fisher PLSD).

#### mRNA Expression Levels of Key Elements of the GABAergic and Glutamatergic Systems in the Basolateral Amygdala

Finally, we investigated the expression levels of genes encoding key elements of the GABAergic synapses, which are the GABA-producing enzyme (Gad67), vesicular GABA transporter (Vgat), and parvalbumin (Pvalb). Additionally, we measured one glutamatergic marker, vesicular glutamate transporter (Vglut), in the basolateral amygdala.

**Figure 8:**
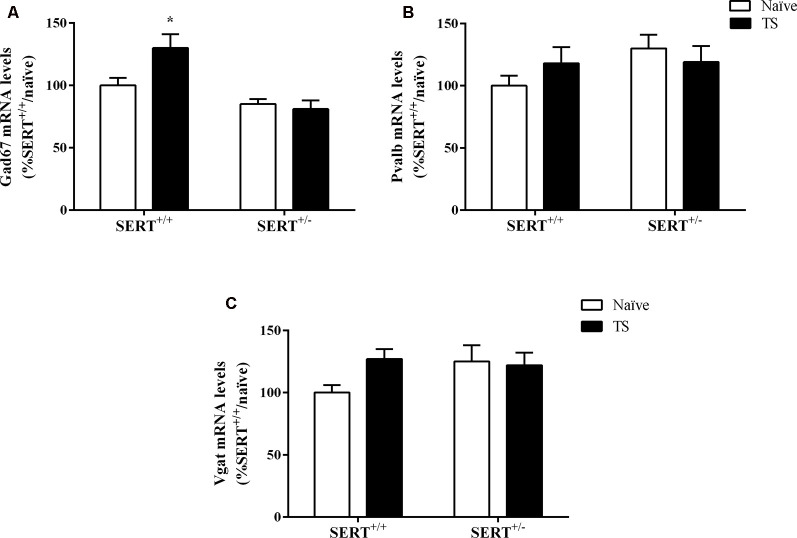
Gad67 **(A)**, Parvalbumin (Pvalb; **B**) and vesicular GABA transporter (Vgat; **C**) mRNA levels in the basolateral amygdala of heterozygous serotonin transporter knockout (SERT^+/–^) and wild-type (SERT^+/+^) male rats exposed to TS from postnatal day 8–14 or a control treatment (no TS-naïve). Data are expressed as a percentage of naïve SERT^+/+^ (set at 100%) and are represented as mean ± SEM. *Significantly different from naïve SERT^+/+^ rats (*p* < 0.05).

Two-way ANOVA showed a significant effect of genotype (*F*_(1,33)_ = 17.158, *p* = 0.000) and an interaction between genotype × TS (*F*_(1,33)_ = 4.971, *p* = 0.033) for Gad67 gene expression (Figure 8A). We found a significant increase in its mRNA levels specifically in TS SERT^+/+^ animals in comparison to naïve SERT^+/+^ rats (+30%, *p* = 0.007, Fisher PLSD). We did not observe any alteration in Pvalb mRNA levels and Vgat mRNA levels (Figures 8B,C).

**Figure 9:**
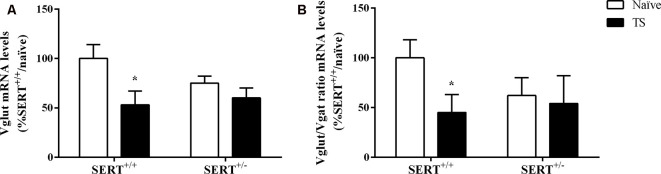
Vesicular glutamate transporter (Vglut; **A**) and the ratio between Vglut and vesicular GABA transporter (Vgat; **B**) mRNA levels in the basolateral amygdala of heterozygous serotonin transporter knockout (SERT^+/–^) and wild-type (SERT^+/+^) male rats exposed to TS from postnatal day 8–14 or a control treatment (no TS-naïve). Data are expressed as a percentage of naïve SERT^+/+^ (set at 100%) and are represented as mean ± SEM. *Significantly different from naïve SERT^+/+^ rats (*p* < 0.05).

As shown in Figure 9A, we observed a significant effect of TS on Vglut gene expression (*F*_(1,33)_ = 7.071, *p* = 0.015, two-way ANOVA). TS SERT^+/+^ animals showed a decrease in its mRNA levels (−47% vs. naïve SERT^+/+^, *p* = 0.009, Fisher PLSD).

Finally, we calculated the Vglut/Vgat ratio. Two-way ANOVA revealed that there was a trend effect for TS on this ratio (*F*_(1,32)_ = 3.812, *p* = 0.061). Further, *post hoc* testing revealed that the ratio was significantly reduced in TS SERT^+/+^ rats compared to naïve SERT^+/+^ rats (−55%, *p* = 0.047, Fisher PLSD; Figure 9B).

## Discussion

In this study, we investigated the effect of neonatal TS in male SERT^+/–^ rats on social and affective behavior, as well as gene expression in the basolateral amygdala as readouts. By applying TS 1 × day/10 min from PND 8–14 we observed that diminished social contact and increased non-social behavior in naïve SERT^+/–^ rats were normalized in TS SERT^+/–^ animals. Also, we observed that increased anxiety as measured in the elevated plus-maze test and reduced anhedonia as measured in the 3% sucrose consumption test was normalized in TS SERT^+/–^ rats, suggesting that neonatal TS had a beneficial influence on the development of the effective behavior in SERT^+/–^animals. Interestingly, at the molecular level, we observed a strong effect of TS on mRNA expression levels of genes encoding key elements of the Bdnf, GABA and glutamate systems, and on mRNA expression levels of genes encoding glucocorticoid-responsive genes in the basolateral amygdala of SERT^+/+^ but not SERT^+/–^ animals.

Previous work has demonstrated that inherited down-regulation of SERT is associated with increased sensitivity to the adverse effects of early life stress, resulting in increased anxiety- and depression-like behavior (Houwing et al., 2019). However, the SERT does not only increase sensitivity to negative environmental stimuli, since this would preclude the existence of the high frequency of the 5-HTTLPR in the human population. Given that, it is unlikely that a so common gene variance is maintained throughout evolution only exerting negative effects. Indeed, there is evidence, particularly from human studies, that the 5-HTTLPR also increases sensitivity to positive environmental stimuli, in line with the differential susceptibility theory (Belsky et al., 2009). TS is a positive manipulation that when applied during neonatal periods can prevent the development of anxiety and depression (Boufleur et al., 2012; Freitas et al., 2015). Accordingly, we observed that naïve SERT^+/–^ rats displayed increased anxiety in the elevated plus-maze test and that this behavior was normalized in TS SERT^+/–^ rats. Also, we observed a reduction in sucrose preference and intake in naïve SERT^+/–^ animals in the sucrose 3% preference test, indicative of an anhedonic state in the animals, while TS SERT^+/–^ animals showed a normalization of the sucrose preference up to the level of SERT^+/+^ rats. Based on these findings we can hypothesize that animals submitted to TS did not develop the anxiety- and anhedonia-like behaviors as observed in naïve SERT^+/–^ animals. Since we did not test the influence of negative stimuli on SERT^+/–^ rats, our findings would reflect vantage sensitivity, which reflects a disproportionally increased sensitivity to positive stimuli (Pluess and Belsky, 2013).

According to literature, SERT^+/–^ animals present an increase in social avoidance after stress exposure (Bartolomucci et al., 2010). Here, naïve SERT^+/–^ animals showed a reduction in social contact time and an increase in non-social behavior time, while TS normalized these observations in SERT^+/–^ animals. Sociability has been strongly associated with amygdala function (Hitti and Siegelbaum, 2014) since the neuroplastic changes accompanying the social decisions in rodents involves modulation of the amygdala. For instance, amygdala lesions have been related to alterations in social behavior in juvenile and adult rats (Daenen et al., 2002) and changes in amygdala circuits also have been associated with sociability deficits and anxiety symptoms in mice (Li et al., 2019).

At the molecular level, in general, we did not find significant alterations in naïve SERT^+/–^ rats compared to naïve SERT^+/+^ rats, while in TS SERT^+/+^ animals there were basolateral amygdala modifications when compared to naïve SERT^+/+^ animals. Possibly, SERT^+/+^ animals exhibit a large dynamic space for adjustments to buffer environment influences while SERT^+/–^ animals may lack such a dynamic space because of a tonic elevation in neuronal activity. This may render them susceptible to environmental influences, including supportive stimuli (Homberg et al., 2016). A previous study reported that in SERT^−/−^ mice, compared to wild-type mice, spine density in the amygdala was significantly increased. Interestingly, after stress exposure, behavioral changes were observed selectively in the SERT^−/−^ mice, while spine density remained unaltered in these mice. In wild-type controls, spine density increased up to the level of that of SERT^−/−^ mice. These findings suggest that a tonic increase in excitability reduces plasticity when a further increase in excitability is required to process the stress, failing to buffer the effects of stress and exaggerated behavioral stress response (Nietzer et al., 2011). It is plausible that a comparable mechanism is at play in the present study, with SERT^+/–^ rats responding behaviorally to TS due to a lack of a dynamic range to molecularly “neutralize” the effects of TS.

Previous works demonstrated that Bdnf levels were reduced in the prefrontal cortex and hippocampus of SERT^−/−^ animals throughout life (Molteni et al., 2010; Calabrese et al., 2013). Bdnf is a key player in neurodevelopment and neuronal plasticity. Here, we observed a decrease in the expression of total Bdnf and its isoform IV and VI in TS SERT^+/+^ animals compared to naïve SERT^+/+^ rats. Although an increase in Bdnf expression in the brain is generally related to an antidepressant effect, Bdnf has been reported to have an opposite role in the amygdala. Indeed, overexpression of Bdnf in the amygdala has been related to an anxiogenic response (Govindarajan et al., 2006). Also, a Bdnf up-regulation in the central amygdala of SERT^−/−^ rats is related to enhance negative emotional state, contributing to a compulsive drug self-administration behavior (Caffino et al., 2019). Not only the total Bdnf but also alterations in Bdnf isoform VI and IV were related to increased anxiety in male rats after acute stress exposure (Luoni et al., 2016; Pandey et al., 2017). Although a previous study demonstrated that TS in Wistar rats led to an increase in Bdnf levels in the hippocampus, along with a beneficial effect on anxiety and an improvement in working memory as evaluated using the Y-maze test (Antoniazzi et al., 2017), in our study the decrease of Bdnf expression in the basolateral amygdala in TS animals could be indicative of a protective mechanism against anxiety and anhedonic behaviors.

To assess if the HPA axis is modulated by TS in SERT animals, we investigated the expression of both glucocorticoid (Nr3c1) and mineralocorticoid (Nr3c2) receptor encoding genes. There was an increase in Nr3c2 gene expression only in TS SERT^+/–^ animals compared to TS SERT^+/+^ animals and there were no changes in Nr3c1 gene expression. Nonetheless, the ratio of Nr3c1/Nr3c2 was found to be increased in the basolateral amygdala of both SERT^+/+^ and SERT^+/–^ TS exposed animals. In previous work, this ratio was found to be reduced in the paraventricular nucleus and hippocampus of stressed animals (Brydges et al., 2014; Murgatroyd et al., 2015). The ratio between glucocorticoid and mineralocorticoid expression plays a key role in the promotion of health, homeostasis, and adaptation (de Kloet et al., 1993). An increase in this ratio as observed in TS SERT^+/–^ animals could indicate a change in homeostasis, leading to neuroadaptation and improving the ability to cope with different environmental situations and explaining the normalization of anxiety behavior observed in this group. Furthermore, we measured expression levels of glucocorticoid-regulator FK506-binding protein 51 (Fkbp5), Nr4a1, and growth arrest and DNA damage-inducible factor 45 (Gadd45β). We observed that FKBP5 was increased in TS SERT^+/+^ animals compared to naïve SERT^+/+^. Literature is controversial regarding Fkbp5 expression in the brain. Some studies showed increased expression levels of Fkbp5 in the basolateral amygdala of stressed rats (Xu et al., 2017) as well as increased FKBP5 mRNA in the ventral prefrontal cortex of homozygous SERT rats after early life stress exposure (van der Doelen et al., 2014). In contrast, maternal separation reduced Fkbp5 expression in the hippocampus and did not alter in the amygdala of adult male mice (Candemir et al., 2019). Furthermore, we observed that Nr4a1 expression was reduced in both TS manipulated SERT^+/+^ and SERT^+/–^ animals. Nr4a1 is an activity-dependent immediate early-gene responding to a variety of sensory stimuli, adapting the synaptic activity in response to stimuli. Prolonged expression of Nr4a1 can lead to mitochondrial malfunction and altered synaptic plasticity (Chen et al., 2014; Jeanneteau et al., 2018). Accordingly, the reduction of Nr4a1 expression in TS groups could explain the mechanism of protection against brain disturbances. Moreover, Gadd45β has been associated with amygdala-related learning tasks and the epigenetic programming of social behavior (Kigar et al., 2015). Despite Gadd45β’s effects on social behavioral function, we did not observe a change in Gadd45β expression in both naïve and TS SERT^+/–^ animals. Only in TS SERT^+/+^ animals, we found a change, possibly contributing to the maintenance of brain homeostasis. This could explain why there was no change in behavior, as a response to TS in SERT^+/+^ rats.

The basolateral amygdala is a brain region involved in the processing of emotional signals and contains GABAergic interneurons. The amygdala undergoes developmental changes in early life and is fully mature around adolescence. Environmental events during early life or the developmental stage of the amygdala can have long-lasting persisting effects (Bessières et al., 2019). The balance between inhibitory and excitatory neurotransmission is necessary for brain development (Chamberland and Topolnik, 2012). Here, we investigated the expression levels of Gad67, Pvalb, vesicular GABA transporter (Vgat), and vesicular glutamate transporter (Vglut) to reflect the excitatory/inhibitory balance in the basolateral amygdala. We observed an increase in the GABAergic marker Gad67 in TS SERT^+/+^ rats compared to naïve SERT^+/+^ rats. Gad67 serves as an inhibitory marker that helps in the GABA synthesis under basal conditions. Stress is related to decreases in Gad67 expression in the medial prefrontal cortex, impairing social behavior in rats, and reducing inhibitory synapses in the brain (Ohta et al., 2019). Evidence has shown that treatment with the antidepressant duloxetine can restore the reduction of Gad67 in stress-depressive state animals (Guidotti et al., 2012). Moreover, inhibitory and excitatory amygdaloid circuits were found to be affected in patients with depression, bipolar disorder, or schizophrenia, which was paralleled by a decrease in GAD67 and an increase in VGLUT levels in various nuclei of the amygdala in all patients (Varea et al., 2012). The increase in Gad67 observed in TS SERT^+/+^ rats may suggest that the manipulation increased the number of GABAergic terminals in the basolateral amygdala, preventing anxious-depressive behaviors. Regarding excitatory neurotransmission, TS SERT^+/+^ animals showed a reduction in both Vglut expression levels and Vglut/Vgat ratio. Stress has been associated with enhancement of glutamatergic neurotransmission in prefrontal cortex and amygdala, and peripubertal stress has been related to increasing the Vglut/Vgat ratio, which in turn was associated with the development of psychiatric disorders (Yuen et al., 2011; Tzanoulinou et al., 2014). Although we did not find alterations in SERT^+/–^ groups, the maintenance of normal anxiety behavior observed in TS SERT^+/+^ animals could be explained by the reduction in this glutamatergic expression, in which these reduced levels could prevent sensitivity to adversity in these animals.

Summarized, our findings indicate that exposure to neonatal TS in SERT^+/–^ and SERT^+/+^ animals result in lasting changes in emotional and social parameters and molecular changes in the basolateral amygdala. Generally, the data suggest that TS in SERT^+/–^ animals had pronounced effects on anxiety and social behaviors, but not on gene expression in the basolateral amygdala, possibly because of SERT^+/–^ animals present at baseline a tonic elevation in neuronal activity, hindering further changes in gene expression upon environmental challenges. On the other hand, TS in SERT^+/+^ animals altered molecular parameters in the basolateral amygdala but these effects were not accompanied by behavioral changes, suggesting that SERT^+/+^ animals might have a greater dynamic range for adjustments, allowing them to remain unaffected by TS. One limitation of the present study is that we did not include female rats. Since women show more susceptibility to depression (Kuehner, 2017), studies comparing male and female are still needed to better understand the relation between TS and SERT^+/–^ animals in both sexes. To conclude, this is the first study to investigate the beneficial effects of an early-life supportive environmental stimulus on later life behavior of stress-sensitive SERT^+/–^ animals, with results that will further our understanding of how a supportive environment particularly benefits vulnerable individuals.

## Data Availability Statement

The raw data supporting the conclusions of this article will be made available by the authors, without undue reservation.

## Ethics Statement

The animal study was reviewed and approved by Committee for Animal Experiments of the Radboud University Nijmegen, The Netherlands.

## Author Contributions

KR and CB experimented and collected the behavioral data. CB, FC, and PB performed and analyzed the molecular data. KR analyzed the data and wrote the manuscript. JH, MV, CA, MB, and KR designed the study and interpreted the data. JH, MV, and MB reviewed and edited the manuscript. JH and MR were responsible for the funding acquisition. All authors contributed to the article and approved the submitted version.

## Conflict of Interest

The authors declare that the research was conducted in the absence of any commercial or financial relationships that could be construed as a potential conflict of interest.
